# Measurement of Tumor Pressure and Strategies of Imaging Tumor Pressure for Radioimmunotherapy

**DOI:** 10.1007/s13139-019-00598-7

**Published:** 2019-06-13

**Authors:** Hyeon-gi Kim, A. Ram Yu, Jae Joon Lee, Yong-Jin Lee, Sang Moo Lim, Jin Su Kim

**Affiliations:** 1 Division of RI application, Korea Institute of Radiological and Medical Sciences & Radiological and Medico-Oncological Sciences, University of Science and Technology (UST), 75 Nowon-Gil, Gongneung-Dong, Nowon-Gu, Seoul, 01812 South Korea; 2 Laboratory Animal center, Osong Medical Innovation Foundation, Cheongju, Chungbuk South Korea; 3 Department of Nuclear Medicine, Korea Institute of Radiological and Medical Sciences, Nowon-gu, South Korea; 4 Radiological and Medico-Oncological Sciences, University of Science and Technology (UST), Seoul, South Korea

**Keywords:** Tumor pressure, Monoclonal antibody, Radioimmunotherapy, ADC

## Abstract

Tumor interstitial pressure is a fundamental feature of cancer biology. Elevation in tumor pressure affects the efficacy of cancer treatment and results in the heterogenous intratumoral distribution of drugs and macromolecules. Monoclonal antibodies (mAb) play a prominent role in cancer therapy and molecular nuclear imaging. Therapy using mAb labeled with radionuclides—also known as radioimmunotherapy (RIT)—is an effective form of cancer treatment. RIT is clinically effective for the treatment of lymphoma and other blood cancers; however, its clinical use for solid tumor was limited because their high interstitial pressure prevents mAb from penetrating into the tumor. This pressure can be decreased using anti-cancer drugs or additional external therapy. In this paper, we reviewed the intratumoral pressure using direct tumor-pressure measurement strategies, such as the wick-in-needle and pressure catheter transducer method, and indirect tumor-pressure measurement strategies via magnetic resonance.

## Introduction

Tumor interstitial pressure is a fundamental feature of cancer biology. Elevated tumor pressure affects the efficacy of other cancer treatments as well by causing a heterogeneous intratumoral distribution of drugs and macromolecules [1–3]. Monoclonal antibodies (mAb) play a prominent role in cancer therapy. For instance, mAb enhances the immune response of a patient via various mechanisms by interacting with specific cancer-cell antigens. It also inhibits cell growth factors, thereby preventing the proliferation of tumor cells. Meanwhile, radionuclide-labeled mAb, also known as radioimmunotherapy (RIT), is an effective form of cancer therapy in which tumor-associated mAb is paired with cytotoxic radionuclides. These radiation-linked mAbs can then be selectively attached to tumor antigens such that cytotoxic radiation can be directly delivered to the tumor [4–7]. RIT has shown clinically significant efficacy for lymphoma and other blood cancers but unfortunately, it has not been successful for solid tumors. This is largely because the high interstitial pressure in the region of solid tumors prevents mAb from penetrating into the tumor [8, 9] The interstitial pressure within the tumor region can be decreased, however, by the use of anti-cancer drugs (e.g., paclitaxel) or additional external therapy (i.e., high focused ultrasound) [9, 10].

## Tumor Microenvironment and Pressure

Although seemingly insignificant in biological systems, pressure is an important determinant to understanding tumor microenvironments, which can influence various factors related to the tumor. Compared with normal tissue, tumors have an abundant extracellular matrix (ECM). This ECM molecularly supports tumor growth and survival [11]. Most strikingly, tumor-associated abundant ECM increases the interstitial fluid pressure (IFP) compared with that of a normal tissue. High-IFP environments are one of the major factors inhibiting the penetration of macromolecules, such as chemotherapy molecules. Moreover, the ECM creates a tumor microenvironment favorable of cancer-cell migration, tumor growth, and resistance to apoptosis. Although it is not clear how tumor-associated ECM increases the pressure [11], various studies have demonstrated that ECM-associated submolecules correlate with the pressure level. Furthermore, high IFP coincides with increased recurrence rates and poor prognoses [12, 13].

Before discussing the IFP in tumors, it is important to consider its physical and theoretical meaning. The relationship between IFP and flow (*J*_*S*_) is given by the Staverman-Kedem-Katchalsky equation [14] or:

$$ {J}_S= PA\left({c}_{\mathrm{V}}-{c}_{\mathrm{i}}\right)+{J}_F\left(1-{\sigma}_F\right)\Delta {c}_{lm} $$

where the first term represents a diffusive component and the second a convective component. In the diffusive component, *P* is the diffusive vascular permeability, *A* is the vessel surface area, and *c*_V_ and *c*_i_ are the plasma and interstitial concentrations of the solute, respectively. In the convective component, *σ*_*F*_ is the solvent-drag reflection coefficient representing the coupling between fluid and solute and Δ*c*_*lm*_is the log-mean concentration calculated by:

$$ \Delta {c}_{lm}=\frac{c_{\mathrm{V}}-{c}_{\mathrm{i}}}{\ln \left({c}_{\mathrm{V}}/{c}_{\mathrm{i}}\right)} $$

where all factors are as previously defined. *J*_*F*_ in the convective component represents the pressure-driven fluid flux across the vessel wall described by Starling’s hypothesis,

$$ {J}_F={L}_PA\left[\left({p}_{\mathrm{v}}-{p}_{\mathrm{i}}\right)-\sigma \left({\pi}_{\mathrm{v}}-{\pi}_{\mathrm{i}}\right)\right] $$

where *L*_*P*_ is the filtration coefficient (or hydraulic conductivity) of the vessel, *A* is the surface area of the vessel, *p*_v_ and *p*_i_ are the vascular and interstitial fluid pressures (*P*), respectively, and *π*_v_ and *π*_i_ are the colloid oncotic pressures in plasma and interstitial fluid, respectively. *σ* is osmotic reflection coefficient.

As proposed by Starling, the plasma oncotic pressure normally exceeds that of the interstitium, largely counterbalancing the hydrostatic forces that typically favor the movement of fluid out of the vasculature and into the interstitium [15]. These relationships, however, depend on the tumor type. For example, they appear to be inverted for pancreatic ductal adenocarcinomas (PDA) for two main reasons. First, if *p*_i_ greatly exceeds *p*_v_, the hydrostatic gradient collapses. Indeed, the findings of Provenzano et al. [16] suggest that in the case of PDA, *p*_i_ is sufficiently high to collapse the vessel itself. Second, the difference between the colloid oncotic pressures in plasma and interstitial fluid (i.e., *π*_v_ and *π*_i_) was very small in solid tumors and as such, *π*_v_ − *π*_i_ can be neglected [17]. Furthermore, the osmotic reflection coefficient, *σ*, is close to 1 for macromolecules and approaches zero for small molecules, suggesting that osmotic pressure gradients are not strong determinants of conventional chemotherapy distribution.

One of the key factors to understanding pressure in biological systems was solid stress (SS). In a seminal study, Jain et al. concluded that proliferating tumor cells led to increased SS [18]. Jain et al.’s work measured SS by cutting the tumor and measuring the tumor opening time [19]. Despite the simplicity of this method, it remains a powerful tool to measure the SS in cancer. Moreover, SS correlates with the density of tissue components like collagen and hyaluronan in the ECM.

Studies aimed at changing the IFP demonstrated that it does not affect SS [19]. Nieskoski et al. demonstrated, however, that there exists a relationship between IFP, SS, and total tissue pressure (TTP or Total IFP) [20]. Meanwhile, TTP comprises SS based on tumor-associated ECM, interstitial stress based on tissue components, cell density based on cellular migration and proliferation, and IFP within the tissue interstitium. Thus, TTP is defined as:

$$ \mathrm{TTP}=\mathrm{IFP}+\mathrm{SS}. $$

**Fig. 1:**
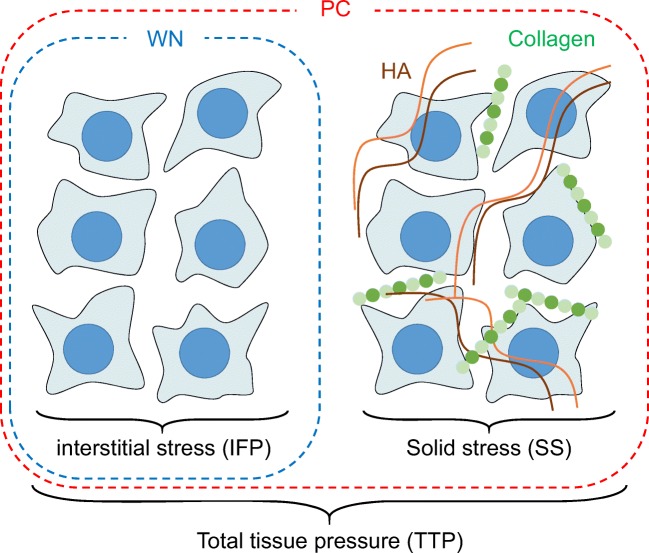
Cartoon representation of the total tissue pressure. The wick-in-needle (WN) method can only measure the interstitial stress. The pressure catheter (PC) method, however, can measure total tissue pressure (interstitial stress + solid stress)

Figure 1 shows cartoon representation of the total tissue pressure. The wick-in-needle (WN) method can only measure the interstitial stress. The pressure catheter (PC) method, however, can measure total tissue pressure (interstitial stress + solid stress).

## Methods for Measuring Pressure

Pressure-detection methods can be classified into two categories: direct and indirect. Direct methods measure the magnitude of the pressure directly while indirect methods measure some quantitative value from which the pressure can be obtained by using a computational algorithm or scaling factor.

### Direct Measurement Methods

**Fig. 2:**
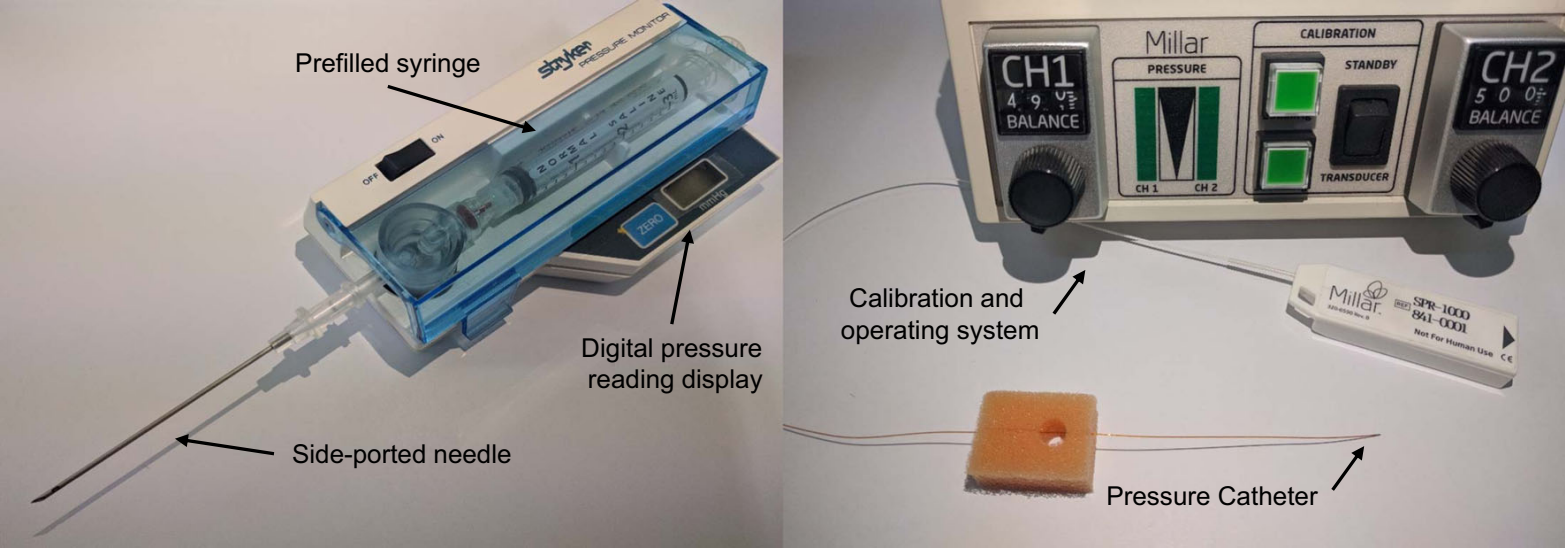
Typical pressure-measurement systems

Figure 2 shows the typical pressure-measurement systems. The classical measurement of pressure involves directly inserting a measurement probe into the tumor tissue or the desired sample. Wick-in-needle (WN) and pressure catheter (PC) are the two most commonly used methods to measure pressure directly.

The WN method is an effective method of measuring IFP for various types of cancer. The wick catheter techniques proposed by Scholander, Hargens, and Miller [21] and Fadnes et al. [22] improved upon the WN method. Nevertheless, it is still limited by the fact that it can only measure IFP, i.e., it cannot be used to measure SS-induced pressure. The SS, however, is important for determining the IFP of tumors exactly.

The pressure catheter (PC) method was developed to overcome this limitation of the WN method. The PC method is an enhanced method of measuring the IFP with high accuracy [23, 24]. Christopher et al. demonstrated that the PC method resulted in a pressure magnitude different from that of the WN method [25]. Namely, the pressure measured by the WN method was less than that of the PC method because the WN method only detected the IFP. To explain the difference between the two methods, we recall the *J*_*F*_ equation.

$$ {J}_F={L}_PA\left[\left({p}_{\mathrm{v}}-{p}_{\mathrm{i}}\right)-\sigma \left({\pi}_{\mathrm{v}}-{\pi}_{\mathrm{i}}\right)\right] $$

Rearranging these terms and solving for *p*_i_ or IFP, gives the following:

$$ {p}_{\mathrm{i}}={p}_{\mathrm{v}}-\sigma \left({\pi}_{\mathrm{v}}-{\pi}_{\mathrm{i}}\right)-{J}_F/{L}_PA. $$

Although transvascular fluid flux exists in most normal tissues, it approaches zero in cancerous tissues (i.e., *J*_*F*_/(*L*_*P*_ *A*) → 0). Substituting this into the above equation, we see that:

$$ {p}_{\mathrm{i}}={p}_{\mathrm{v}}-\sigma \left({\pi}_{\mathrm{v}}-{\pi}_{\mathrm{i}}\right). $$

Jain et al. assumed that *π*_i_ was always less than *π*_v_ and only approached *π*_v_ under conditions of compromised vascular integrity (i.e., *σ* ≅ 0). Under these conditions, the oncotic gradient dissipates, permitting *p*_i_ to equal *p*_v_. With intact vessels, however, *π*_i_ can theoretically surpass *π*_v_ due to the presence of proteins and other oncotically active molecules secreted by the tumor epithelium and stroma [26].

This equation, however, only calculates vascular contributions to the IFP while ignoring the SS, which can affect the IFP in the cancerous tissue. For example, hyaluronic acid (HA) contributes to increasing the IFP in tumors [26–28]. Thus, for tumors:

$$ \mathrm{TTP}={p}_{\mathrm{V}}-\sigma \left({\pi}_{\mathrm{v}}-{\pi}_{\mathrm{i}}\right)+{P}_{\mathrm{i}\mathrm{mmobile}}+{\Pi}_{\mathrm{i}\mathrm{mmobile}} $$

where *p*_V_ − *σ*(*π*_v_ − *π*_i_) describes the Starling forces (or Starling equation) and *P*_immobile_ + Π_immobile_ describes the fluid pressure based on the SS-induced pressure from the hydrostatic and oncotic components [26]. Meanwhile, *P*_immobile_ is biologically significant in describing the pressure from the HA. This term is based on an elastic recoil component and the electrostatic repulsion of negative charges on HA, which contributes to its tendency for expansion [26]. Moreover, Π_immobile_ indicates Donnan potential and van ′t Hoff forces. Thus, the combination of these terms fully represents TTP [26].

### Indirect Measurement Methods

Direct measurement methods are applicable to cancerous or healthy tissues; however, they only measure the pressure at one point and result in damage to the sample. Imaging-based methods of pressure measurement represent one approach to overcoming these limitations. In this chapter, we describe two recent developments in the use of magnetic resonance imaging (MRI) for the indirect measurement of IFP: the apparent diffusion coefficient and convection MRI.

**Fig. 3:**
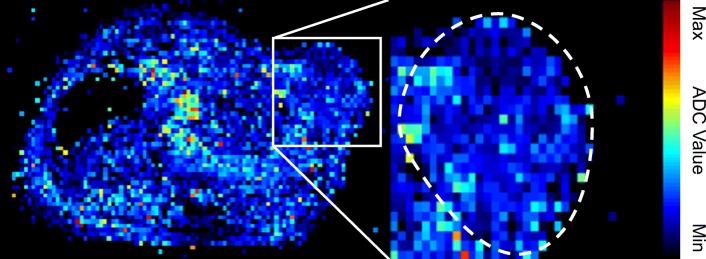
Representative example of an apparent diffusion coefficient (ADC) map. Image of a mouse bearing NCI-N87 human gastric cancer

Diffusion-weighted MRI (DW-MRI) is a powerful imaging technique that is sensitive to the Brownian motion (e.g., molecular diffusion) of water in tissues [29, 30] and provides an apparent diffusion coefficient (ADC) map. Generally, molecular Brownian motion in vivo systems is restrained by ECM, cell population, and organelles. Therefore, ADC maps or values can provide information on the microenvironment or component [31, 32]. Figure 3 shows an example of an ADC map. However, Hompland et al. showed that ADC values have a correlation with the cell density [31]. These studies show that high cell density or living tissue yields low ADC values while high ADC values are measured in a necrotic tissue or an apoptosis tissue [31–33].

Thus, the ADC values resulting from the detection of Brownian motion inversely correlated with cell density because the Brownian motion was limited by the tumor microenvironment.

Furthermore, ADC is related to IFP. Researchers have demonstrated that ADC values are inversely correlated with IFP, using the PC method [31]. Moreover, Hauge et al. demonstrated that the abundance of collagen fiber and ADC have an inverse correlation with IFP [34]. These works strongly suggest that ADC is significant for the understanding of IFP.

However, recent studies have proposed a new technique, known as convection MRI, which measures low-velocity fluid flow in tumors [35]. In this study, the following assumptions are made regarding the convection MRI method: (1) the intravascular MR signal can be made insignificant by dual inversion pulses, (2) the velocity of molecular encoding is sufficiently sensitive to measure the velocity of fluid flow in the interstitium, and (3) the influence of nulled fluid exchange between intra- and extravascular compartments is negligible [35]. Thus, this study demonstrates that the fluid velocity maps can be calculated using computational algorithms to obtain information on the intratumor pressure using convection MRI method [35]. Although classical MR-based ADC maps only represent the Brownian motion, convection MRI data represent 3D pressure and fluid-velocity maps, thereby enhancing the understanding of the influence of cancer on pressure [35].

## Evidence of the Clinical Significance of Pressure

Recent studies have shown that high IFP is correlated with poor response of various tumors to treatment [36–40]. Clinical studies on patients with various types of cancer revealed that the patients who responded best to chemotherapy showed a progressive lowering of the tumor IFP while the tumor IFP of patients who did not respond increased during the course of the treatment [38]. These results show that the IFP was an important predictor of clinical response. Jain et al. demonstrated that IFP directly influenced drug transport [40–42]. Furthermore, ECM molecules [40] and cell-to-cell junctions [43] led to increased IFP in tumor tissues. Several types of treatment, however, have shown efficacy in decreasing tumor IFP and have enhanced the treatment response in animal models and patients.

### Vascular Endothelial Growth Factor Inhibitors

Jain et al. demonstrated that VEGF-receptor inhibition improved the transvascular pressure gradient and enhanced chemotherapeutic drug delivery [44]. Additionally, recent studies have shown that anti-VEGF treatment of patients with rectal carcinomas led to a significant reduction of tumor IFP [45] (Table 1). The normalization of vessels contributes to this decrease in IFP but still does not clearly explain the mechanism. Heldin et al. explained that the normalization of tumor vessels likely decreased the vessel permeability and thereby lowered tumor IFP [11]. Considered together, the recent experiments with VEGF inhibitors suggest that increased vascular permeability is an important contributor to increased tumor IFP [11].

**Table 1:** Drugs known to reduce tumor IFP

Target	Drug or method	Description	Ref
HA	PEGPH20	Degradation of HA	[16]
Angiotensin receptor	Losartan	Inhibition of HA and collagen	[41]
Collagen	Collagenase	Degradation of collagen	[47]
VEGFR	Bevacizumab	Normalization of blood vessels, improved blood flow	[46]

### Pegylated Human Recombinant Hyaluronidase Alfa

Hyaluronic acid (HA) is one component of the ECM. Many common solid tumors have abundant HA in addition to the increased SS and IFP [16]. This molecule can store water in the ECM, which leads to increased pressure. Provenzano et al. demonstrated that pancreatic ductal adenocarcinomas (PDA) maintained a pressure higher than that of normal pancreatic tissue by measuring the IFP using the catheter method [16]. Furthermore, the chemotherapeutic drug gemcitabine has been ineffective in PDA orthotropic tissues because of poor drug delivery caused by high IFP. In order to overcome this problem, Provenzano et al. used the PEGPH20 inhibitor, pegylated human recombinant hyaluronidase alfa. After PEGPH20 treatment, HA abundance and IFP simultaneously decreased in the tumor and the gemcitabine antitumor effect was enhanced beyond the control. These results suggest that the decreased pressure allowed chemotherapy molecules to reach the tumor. The median overall survival increased from 55.5 for Gem+Placebo to 91.5 days for Gem+PEGPH20 [16].

### Collagenase

Collagen is another major component in the ECM along with HA. Similarly, collagen often exists at increased abundance in solid tumors [46]. Collagen affects epithelial-mesenchymal transitions and tumor growth. Eikenes et al. demonstrated that treatment with collagenase reduced the IFP and enhanced drug transport in osteosarcoma (OS) xenograft models [46]. Specifically, collagenase reduced the tumor-associated collagen, which led to a reduction of the IFP by 40% compared with controls as measured by the WN method. Furthermore, fluorescent-labeled TP3 antibody uptake in the tumor increased by a factor of 2 compared with controls. This work suggests that there exists a correlation between pressure and collagen abundance. However, this phenomenon may depend on the cancer type. Jain et al. [41] demonstrated that orthotropic AK4.4 pancreatic tumors do not exhibit a significant correlation with collagen abundance and IFP.

### Angiotensin Inhibitors

Chauhan et al. demonstrated that angiotensin inhibition enhances drug delivery and potentiates chemotherapy [41]. Specifically, they targeted the reduced SS in the tumor with losartan (Cozaar). Losartan is a commonly used treatment for high blood pressure (hypertension). Researchers focused on off-label uses of losartan which demonstrated that it also reduces tumor-associated collagen and hyaluronan production [47]. This work strongly suggests that SS was impacted, thus enabling chemotherapy molecules to reach the tumor.

## Conclusion

Although pressure is a simple physical value, its biological meaning is profound. For example, radioimmunotherapy (RIT) is a powerful method of targeted therapy. Radio-labeled or radio-conjugated macromolecules, however, are restricted by the high IFP in solid tumors. Reducing the IFP is one possible solution to this problem. In the future, methods of reducing the IFP will contribute to improved clinical approaches involving RIT in common solid tumors. Recent developments in IFP measurement methods have enhanced the accuracy and enabled the avoidance of tissue damage. Imaging-based IFP measurement methods will facilitate clinical decision-making in the foreseeable future.
